# Isolation and characterization of nine polymorphic microsatellite markers for the deep-sea shrimp *Nematocarcinus lanceopes* (Crustacea: Decapoda: Caridea)

**DOI:** 10.1186/1756-0500-6-75

**Published:** 2013-03-01

**Authors:** Johannes Dambach, Michael J Raupach, Christoph Mayer, Julia Schwarzer, Florian Leese

**Affiliations:** 1 Zoologisches Forschungsmuseum Alexander Koenig, Bonn, Germany; 2 Deutsches Zentrum für Marine Biodiversitätsforschung, Senckenberg am Meer, Wilhelmshaven, Germany; 3 Department of Animal Ecology, Evolution and Biodiversity, Ruhr University Bochum, 44801, Bochum, Germany

**Keywords:** *Nematocarcinus lanceopes*, Antarctic, Deep sea, Microsatellites, Southern ocean

## Abstract

**Background:**

The shrimp *Nematocarcinus lanceopes* Bate, 1888 is found in the deep sea around Antarctica and sub-Antarctic islands. Previous studies on mitochondrial data and species distribution models provided evidence for a homogenous circum-Antarctic population of *N*. *lanceopes*. However, to analyze the fine-scale population genetic structure and to examine influences of abiotic environmental conditions on population composition and genetic diversity, a set of fast evolving nuclear microsatellite markers is required.

**Findings:**

We report the isolation and characterization of nine polymorphic microsatellite markers from the Antarctic deep-sea shrimp species *Nematocarcinus lanceopes* (Crustacea: Decapoda: Caridea). Microsatellite markers were screened in 55 individuals from different locations around the Antarctic continent. All markers were polymorphic with 9 to 25 alleles per locus. The observed heterozygosity ranged from 0.545 to 0.927 and the expected heterozygosity from 0.549 to 0.934.

**Conclusions:**

The reported markers provide a novel tool to study genetic structure and diversity in *Nematocarcinus lanceopes* populations in the Southern Ocean and monitor effects of ongoing climate change in the region on the populations inhabiting these.

## Findings

The shrimp *Nematocarcinus lanceopes* Bate, 1888 [1] is well-known from the deep sea around Antarctica, the sub-Antarctic islands and the adjacent deep-sea basins of the Southern Ocean [2]. It has a wide vertical distribution from the Antarctic continental slope down to the Southern Ocean abyssal plains at depths of 4,000 m [3]. Previous work using mitochondrial and nuclear sequence data provided clear evidence for a single homogeneous circum-Antarctic *N*. *lanceopes* population [4]. These findings were also supported by species distribution modeling data, showing a large connected habitat around the Antarctic continent and the sub-Antarctic islands [5]. Currently, the Antarctic continent faces in some areas dramatic and most rapid effects of climate change, e.g. atmosphere and ocean warming at the Antarctic Peninsula and in part the sub-Antarctic regions [6]. However, other regions appear quite stable, e.g. eastern Antarctica and the surrounding deep-sea basins [6,7]. The increase in temperature leads to strong declines in winter sea ice extent in the Southern Atlantic and is regarded as a likely cause for the observed dramatic population decline in Antarctic krill populations (see [8]). The decline of this ecological key species has severe impacts on other animals in the Southern Ocean ecosystem that rely on this food source [9]. The consequences of this drastically changing environment on most, in particular benthic deep-sea organisms, are largely unknown and difficult to assess by direct observations. However, the expected responses of species and communities to climate change are critically important for conservation plans and therefore analyses of population processes such as gene flow, local adaptation and loss of genetic diversity are of particular interest (see the *Convention on the Conservation of Antarctic Marine Living Resources*). The reported microsatellite markers allow monitoring the genetic composition of populations, and identifying declines in genetic diversity. Further they enable the identification of genotypes with selective advantages in regions with increased warming or higher rates of primary production [10].

We extracted DNA from pleon muscle tissue using a commercial extraction kit (QIAmp^©^ Blood and Tissue Kit, Qiagen GmbH, Hilden, Germany), following the manufacturer´s protocol. Two different approaches of microsatellite isolation were performed: First we created a small microsatellite enriched library following the Reporter Genome Protocol (RGP) [11]. As a result of this approach we have detected 39 putative microsatellite loci from 96 clone sequences. Out of the 39 tested primer pairs 18 loci amplified successfully. After fragment analysis and testing for polymorphic sites, only one microsatellite marker could be retained due to a lack of polymorphism, stutter bands or unreliable amplification. Therefore, we then used data from a next generation 454 database created in an earlier study to identify putative microsatellite loci in *Nematocarcinus lanceopes* (see [12] for information on library creation and microsatellite statistics). The library was sequenced on a GS-FLX sequencer (Roche, 1/8 of a 454 plate). Coverage estimates for raw reads ranged from 0.11–1.33% [12]. Sequence assembly was performed using the software Geneious 5.5.6. [13]. Microsatellites were detected with the PHOBOS program v3.3.10 integrated as a plugin in Geneious (http://www.ruhr-uni-bochum.de/ecoevo/cm/cm_phobos.htm). Suitable oligonucleotide primers were designed using Primer3 [14] and forward primers tailed with either T7 or Sp6 primer sequence for economic fluorescent labeling of PCR fragments [15]. A “pigtail” sequence was added to problematic reverse primers to reduce stutter bands [16] (see Table 1). We tested 57 primer pairs in the 454 approach whereof eight loci could be retained after testing for polymorphic sites.

**Table 1:** **Characteristics of nine polymorphic microsatellite loci for *Nematocarcinus lanceopes *** **tested on 55 individuals**

**Locus**	**Repeat motif**	**Oligonucleotide primer sequence 5' → 3'**	**Fragment length (bp)**	**N**_**A**_	**H**_**O**_	**H**_**E**_	**F**_**Null Alleles**_
A06	(AG)25	Fw: GTCCTGAGTAATCGGCTCAGCTCT	187–233	17	0.927	0.924	0
		Rev: ACCCAGTTGGAAGCTGTTCTGAG					
NL37	(AAT)19	Fw: GGGTTTAGGAGGAGTTTCGGGAC	186–228	12	0.782	0.832	0.017
		Rev: ACGAGTTGGAATGGGGCTGATG					
NL16	(AG)12	Fw: TCAATTGTCCGGGACGCAAATGT	150–182	13	0.545	0.549	0
		Rev: AGTACATGGGCCACTAACTCCG					
NL44	(ACAG)11	Fw: AATGGAGTGCAATGACGCTTGG	254–328	12	0.818	0.881	0.026
		Rev: TCGCAGTTTGTTTTAGAGGGAGC					
NL51	(AT)12	Fw: TGATGACAGGGATTTGTCTTTCG	144–166	9	0.727	0.824	0.052
		Rev: TCCCCATTTGTACGCTATCC					
NL53	(AC)11	Fw: ACAGTACACAGGCTACATAC	149–179	14	0.800	0.796	0.001
		Rev: ATCTTCATGTTATGCCCTCTAG					
NL55	(AG)11	Fw: ACGCGAACAGTGCTAAGAAGAC	162–216	25	0.927	0.940	0
		Rev: CACCACAGCAAGGAACCTCC					
NL56	(AG)12	Fw: AGTGAAAAGACTCAAATTCCTTGG	151–201	23	0.764*	0.934	0.077
		Rev: CATTACTGCTTCCTTCCTCTC					
NL49	(AG)11	Fw: ACTCTACTTTGGCTTTCTCCCTC	155–183	11	0.927	0.861	0
		Rev: ACACGGGTCTTCCTGAGTGTTG					

*N*_*A *_number of alleles; *H*_*O *_observed heterozygosity; *H*_*E *_expected heterozygosity; *F*_*Null Alleles *_frequency of null alleles.

* Indicates a locus that deviates from HWE after Bonferroni correction (9 comparisons; significance threshold = 0.005). Forward primers of the loci A06, NL44 and NL55 were labelled with a T7 tail (5’-TAATACGACTCACTATAG-3’) all other forward primers were labelled with a Sp6 tail (5’-GATTTAGGTGACACTAT-3’). A “pigtail” (5’-GTTTCTT-3’) was added to the reverse primers of the loci NL49, NL51, NL53, NL55 and NL56 to reduce stutter bands [16].

**Figure 1:**
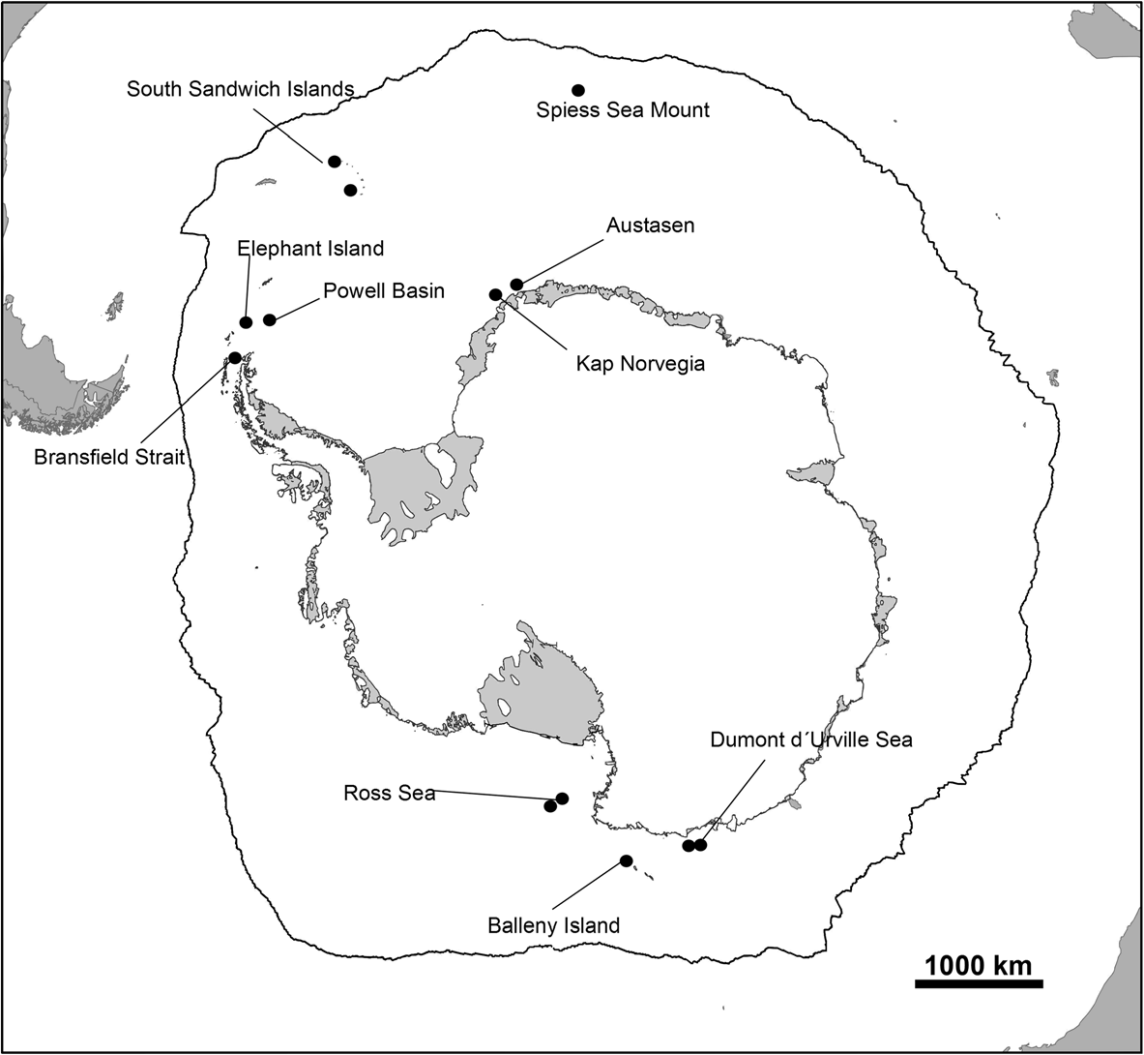
**Sampling sites around Antarctica for specimens of *Nematocarcinus lanceopes *** **Bate,1888 **[1]**.**

Amplification success and polymorphism of the reported microsatellite loci were assessed using 55 individuals of *N*. *lanceopes* from sampling sites all around the Antarctic continent illustrated in Figure 1. PCR amplification of loci was performed with the Qiagen Multiplex PCR kit (Qiagen Inc., USA) in 10 μl reaction volumes using a GeneAmp 2720 Thermo Cycler (Applied Biosystems, USA). The temperature profile for amplification was as follows: (1) initial denaturation at 95°C for 15 min, (2) 20 cycles: 95°C for 60 s, 58°C for 30 s and 72°C for 30 s followed by (3) 10 cycles: 95°C for 60 s, at 53°C for 30 s and 72°C for 30 s and (4) final extension at 72°C for 40 min. We used slightly modified protocols for steps 2 and 3 for amplifying three microsatellite loci: the loci NL55 and NL56 were amplified with annealing temperatures of 60°C (20 cycles) and 55°C (10 cycles) and locus NL44 with annealing temperatures of 55°C (20 cycles) and 53°C (10 cycles). PCR products were multiplexed and added to a mixture of Hi-Di™ formamide and size standard (Gene Scan 500 LIZ) and run on a 3500XL Genetic Analyzer capillary sequencer (Applied Biosystems, USA). To identify the obtained alleles based on the resulting electropherograms, the software GENEMAPPER v4.0 (Applied Biosystems, USA) was used. The software INEst [17] was used to estimate allele frequencies, observed and expected heterozygosity of each locus, and to assess the proportion of possible non-amplifying (“null”) alleles. Of the 96 tested primer pairs, nine loci reliably amplified the desired locus and were polymorphic with 9–25 alleles per locus. The observed heterozygosity (H_O_) ranged from 0.545 to 0.927 and the expected heterozygosity (H_E_) from 0.529 to 0.934. Null allele frequencies ranged between 0 and 0.077. The software GENEPOP v4.1.4 [18] was used to test for deviations from Hardy-Weinberg equilibrium (HWE) and for linkage disequilibrium. After Bonferroni correction for multiple comparisons, one locus showed significant deviation from expectations under HWE (see Table 1). Linkage disequilibrium was not detected for any of the pairwise loci comparisons. Nucleotide sequence information of the described loci is deposited in the Dryad Repository (http://datadryad.org/) and accessible via the link http://dx.doi.org/10.5061/dryad.gg3rn.

The reported set of polymorphic microsatellite loci from *Nematocarcinus lanceopes* will help to analyze the fine-scale genetic structure around the Antarctic continent. These results will help us to test, whether there is regional differentiation that we have not yet detected relying on a single mitochondrial marker only [4], whether gene flow is maintained primarily by the Antarctic Circumpolar Current, whether populations in regions under severe climate change (e.g., the West Antarctica) experience population declines or possibly growth, and whether patterns of local adaptations can be detected in the different regions. The markers will also allow monitoring the effects of the rapid climate change on the population genetic structure of this species.

## Competing interests

All authors declare that they have no competing interest (no financial or non-financial competing interests).

## Authors’ contributions

JD, FL and MJR did the study design and drafted the manuscript. JD, JS and FL carried out the laboratory work. CM, JD and JS carried out the computational work for loci identification and the statistical analysis. All authors wrote the manuscript and all authors read and approved the final manuscript.
